# Language of Administration as a Border: Wild Food Plants Used by Setos and Russians in Pechorsky District of Pskov Oblast, NW Russia

**DOI:** 10.3390/foods10020367

**Published:** 2021-02-08

**Authors:** Olga Belichenko, Valeria Kolosova, Denis Melnikov, Raivo Kalle, Renata Sõukand

**Affiliations:** 1 Department of Environmental Sciences, Informatics and Statistics, Ca’Foscari University of Venice, Via Torino 155, Mestre, 30172 Venice, Italy; valeriia.kolosova@unive.it (V.K.); renata.soukand@unive.it (R.S.); 2 Institute for Linguistic Studies, Russian Academy of Sciences, Tuchkov Pereulok 9, 199004 St Petersburg, Russia; 3 Komarov Botanical Institute, Russian Academy of Sciences, Professor Popov St. 2, 197376 St Petersburg, Russia; DMelnikov@binran.ru; 4 University of Gastronomic Sciences, Piazza Vittorio Emanuele 9, 12042 Pollenzo, Bra (Cn), Italy; raivo.kalle@mail.ee or

**Keywords:** wild food plants, local ecological knowledge, Seto, ethnic minority, post-Soviet ethnobotany

## Abstract

Socio-economic changes impact local ethnobotanical knowledge as much as the ecological ones. During an ethnobotanical field study in 2018–2019, we interviewed 25 Setos and 38 Russians in the Pechorsky District of Pskov Oblast to document changes in wild plant use within the last 70 years according to the current and remembered practices. Of the 71 botanical taxa reported, the most popular were *Vaccinium vitis-idaea*, *Vaccinium oxycoccos*, *Vaccinium myrtillus*, *Betula* spp., and *Rumex acetosa*. The obtained data was compared with that of 37 Setos and 35 Estonians interviewed at the same time on the other side of the border. Our data revealed a substantial level of homogeneity within the plants used by three or more people with 30 of 56 plants overlapping across all four groups. However, Seto groups are ethnobotanically closer to the dominant ethnic groups immediately surrounding them than they are to Setos across the border. Further study of minor ethnic groups in a post-Soviet context is needed, paying attention to knowledge transmission patterns.

## 1. Introduction

While the influence of environmental factors, such as ecosystem changes, on the patterns of wild food plant use are widely acknowledged, the economic and sociocultural factors are less studied yet equally important for the valorization, maintenance, or abandonment of wild foods [1]. What happens to Local Ecological Knowledge (LEK) after a rapid socio-economic shift? While claims of degradation and erosion of LEK have become common place, the exact nature of the change has yet to be described and interpreted. As stated in a recent review [2], modernization, education, and the market are the main drivers triggering changes in LEK. 

A shift to a market economy is perceived differently by poor and more economically stable societies. For the former, wild food plants become an important economic resource [3,4], while for the latter, a trigger for recreational practices and delicacies [5,6]. Food is one of the most important identity markers, so the preference for certain foods, or food plant cultural markers [7], can be regarded as a community-building feature, as can the rejection of certain foods [8]. A study by Quave and Pieroni [9] shows, however, that wild food repertoires in neighboring cultures may converge in the context of food shortages caused by war. 

Wild food is considered a relatively stable area of LEK, more homogenous and less subject to change compared to the medicinal use of plants [9]. However, changes inevitably occur. Classical studies on these changes have been carried out related to migration and the phenomenon is relatively well studied: the extent to which migrants adapt to the food culture of a new host country or maintain their own depends on a wide variety of factors (see [10], and the references therein). However, changes also occur due to changing socio-economic conditions. It has already been shown that in some regions geopolitical changes have led to the diverse evolution of wild food plant consumption; however, this is more difficult to track. We have approached this question by studying ethnic groups divided by a state border. For example, in Bukovina, the border between Romania and Ukraine (since the 1940s), which divided an ethnic group (Hutsuls), created the situation in which the ethnogastronomy of wild plants was more similar to the other ethnic group living within the same country than among Hutsuls themselves [11]. For the Bukovina example, the border was solid, impenetrable, and remains so even now, restricting communication on all levels for the last 80 years, with several generations growing apart. The next issue is to understand how long it actually takes LEK to differ enough to be noticed and whether the separation needs to be ‘solid’.

In order to describe the impact of administrative and later state border division on LEK, we took as a case study the small ethnic group of Seto people, who live on the border between Estonia and the Russian Federation and were a single cohesive group until 1944. According to the linguistic classification, Seto speak a subdialect of Võro dialect of Estonian language, while Setos consider it a language [12]. Between 1920 and 1940, the territory was part of Estonian Republic. As the ethnobotany of Seto has been recently described as a part of the same project [13], we took a comparative case from Pechorsky District of Pskov Oblast of the Russian Federation, a rural area where two ethnic groups, Setos and Russians, have historically resided side by side [14]. This gives us the possibility to compare the influence of administrative and state division, as the Seto community was divided in two by an administrative border in 1944 and later by a state border in 1991. Attracted by Estonian-language education and better economic conditions, the majority of Setos migrated to Estonia leaving less than 200 Setos on the Russian side of the border. In 2010, the Seto were included in the list of indigenous ethnic minorities of Russia.

In spite of numerous works dedicated to various aspects of Seto society and culture, such as ethnography [14,15], demography [16,17], folklore [18] including the traditional style of singing [19], clothing and costumes [20,21], and religion [22,23,24], the ethnobotany of Setos on the Russian side of the border has not yet been described. Some fragments of Seto TEK were provided in quantitative comparative works on Finnic ethnobotany [25,26]. 

The aims of this article are to (1) document current and past practices related to the use of wild food plants in the region, (2) conduct a cross-cultural comparison between Setos and Russians and a cross-border comparison with Setos living on the Estonian side, and (3) outline the factors that have influenced differences and changes in LEK in the region within one generation. Our working hypothesis is that the long-term administrative, and later state-based, division has driven the two Seto groups further apart and closer to their neighboring dominant ethnic and linguistic groups. 

This is the first comprehensive description of the wild food ethnobotany of the Pechorsky District. The research is part of a larger study investigating the effect of centralization on the small ethic groups residing on the borders of former Soviet republics. Cross-border studies of this project have already been reported for Estonian Setomaa (Seto ‘Seto land’) [13], Russian Karelia [27], and Bukovina [11,28].

## 2. Data and Methods

### 2.1. Research Area

Pechorsky District (1251 km^2^) is a subdivision of Pskov Oblast located in North-West Russia on the border with Estonia. The landscape and flora of Pechorsky District differ from that of Pskov Oblast. The study area occupies a part of the Haanja Upland that lies in the Southeast of Estonia and Latvia. The north of the region is dominated by Lake Peipus (3550 m^2^). 

### 2.2. Vegetation of Pechorsky District

The natural conditions around Pechory were thoroughly studied for the first time in the 1920s [29]. According to Tammekan [29], while the Pechory area belonged to the Pskov Governorate, the forests in that area had been rapaciously managed for a long time. Due to irrational forest management, only pine forest fractions were growing in low-fertile areas while very small spruce-birch forests were growing in fertile areas. Intensive management was evident by the fact that the forests were less than 50 years old. Most of the area at that time was arable land or semi-natural meadows [29]. A second comprehensive description of the natural conditions of the area [30] showed that during a 10-year period (1990–2000) arable lands were abandoned and decreased in area from 77% to 60%. Natural meadows (14% of the total arable land), which are mainly located on the banks of water bodies, started to become overgrown with shrubs after the cessation of economic activities. Settlement areas increased the most during those 10 years from 0.6% to 14%. Forested land, which is affected by frequent natural fires in the area, remained about the same at 19%. Pine trees (82%) dominate these forests, with a few other trees present in smaller numbers (8.5% birch and 8% spruce). A decrease in the species richness of common plants was also observed [30]. Vegetation also decreased in the part of Setomaa that remained in Estonia: in 1971 there were 717 species, but 149 of them had disappeared by 2005 [31].

### 2.3. Archaic Seto Culture and Early Contact with Russians

Archaeological, linguistic, and genetic analyses have shown that Finnic tribes came to the Baltic Sea area as early as 3500 years ago [32]. The Setos used to live in a large area along the eastern shore of Lake Peipus, around Lake Pskov and the Velikaya River [33]. After the invasion of East Slavs, the Principality of Pskov was established in the year 862 in the Seto territories. In 1456, Muscovite Russia (the Tsardom of Russia beginning in 1547) conquered the Principality of Pskov, and the area was annexed to the Russian state. The new government began to carry out an extensive Orthodox mission at the border, building Orthodox churches and fortifications (e.g., a monastery in Pechory). In 1772, the Pskov Governorate was formed [34]. Although the Finnic speakers in the Pskov Governorate had been described as early as the 18th century, it was not until the end of the 19th century that Seto culture was scientifically examined by folklorist Jakob Hurt (1839–1906). Thanks to him, other scientists became interested in this ethnic group. Hurt himself studied the Seto for more than 20 years, and his scientific articles and monographs are important early sources [34]. As church services in Pskov province were delivered in Slavonic, which the Setos did not understand, until the end of the 19th century Seto culture preserved archaic pagan features [24,35], which have survived in part to this day. The unique Seto style of singing, *leelo*, was added to the UNESCO World Heritage List of intangible culture in 2009. In 2010, the Seto Institute was established, which coordinates Seto research today. In 2017, all Seto settlements in Estonia were merged under one municipality.

### 2.4. Setomaa and Its Separation 

According to the Tartu Peace Treaty (2 February 1920), small ethnic groups of Finno-Ugric peoples, including the Ingrians and the Setos, were integrated into the Republic of Estonia [29]. Petseri County was formed in the area inhabited by the Setos when settled in Estonia. In 1928, more than 58,000 people lived in the region, including Russians (65%), Setos (25.6%), and Estonians (7.4%), as well as Latvians, Jews, and Germans. In 1922, municipalities were formed along ethnic boundaries, and thus municipalities with only Russian or only Seto residents emerged [29]. However, in 1944, during World War II, a large part (75%) of Petseri County was annexed to the Pskov region of Russia by order of Stalin. This broke the historical boundaries of ethnic Seto settlements. To date, historians have not found any documentation as to why Stalin needed such a border change, which did not follow any ethnic or geographical logic [36]. There is a point of view that the change was based on the higher proportion of ethnic Russians in the annexed territories [37]. One can only assume that Russia wanted to take control of and have authority over the only consistently operating monastery in Pechory. All other monasteries in the USSR had already been closed and/or destroyed before the opening of the Eastern Front of WWII. 

### 2.5. Seto School Education in Pechory

While in Estonia and Livonia the spread of literacy promoted by Lutheran parochial schools began in the 1840s [38], Setos living in the Pskov Governorate received no schooling at all, because schools were not interested in teaching Setos who did not speak Russian, and so only a few Seto men learned to read [35]. It was only during the time of the Republic of Estonia when the Seto began to receive education in Estonian. The first thing which occurred after the War of Independence was that the Seto people were given family names which they did not have before. Village schools were also established in Petseri County and all Setos were provided with school education in Estonian. The first reading book in Seto was published in 1922. Church congregations were also segregated: ethnic Russians were allowed to preach in Russian, and then Seto congregations began to deliver sermons in Estonian [39]. During World War II, when the Seto area was annexed to the Pskov Oblast, all affairs there began to be conducted in Russian However, a network of primary schools with education in Estonian was preserved, and the secondary education was delivered in Secondary School No2 of Pechory, established in 1956, which became the only school in Russia where all subjects were taught in Estonian until the 2004–2005 academic year [40]. It was the lack of an Estonian-language vocational or higher education that was the main reason for the mass immigration of Setos into the territory of Estonian SSR (see Table 1).

### 2.6. Field Data

**Figure 1:**
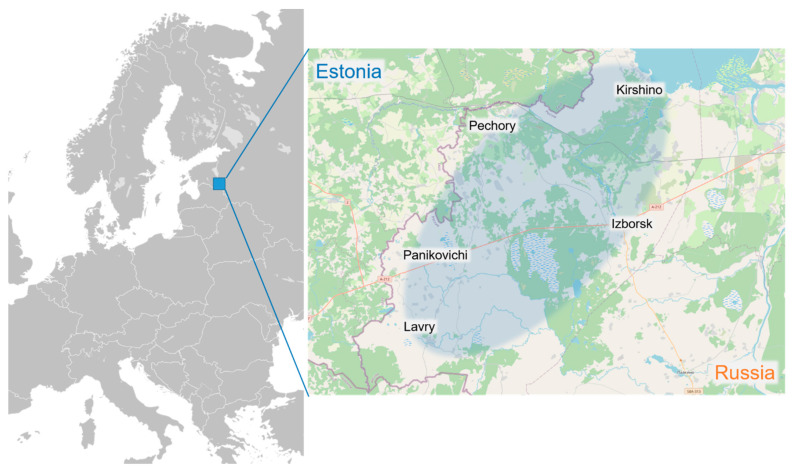
Study area in Pechorsky District of Pskov Oblast. Map base: Wikimedia Commons and OpenStreetMap.org.

The data were collected during the summers of 2018 and 2019 by interviewing the Seto and Russian populations of the Pechorsky District of Pskov Oblast. The informants were recruited using snowball and convenience sampling methods (Setos and Russians, respectively). The interviews were conducted in the villages traditionally inhabited by the two ethnic groups. The majority of the interviews were conducted within the area surrounded by the town of Pechory, and villages of Izborsk, Panikovichi, and Lavry, while three interviews were conducted near Kirshino in Krupp Parish (Figure 1). Five Seto participants were born to mixed families where only one parent was Seto. Among them, only four participants (two families) belonged to a family where both parents were Seto. In such cases, attribution to a group was made on the basis of the language spoken and self-identification of the person as either Seto or Russian. We also interviewed two people who considered themselves Estonian, whom we included in the Seto group, as this ethnonym is still sometimes used by older generations in accordance with the obligatory Soviet attribution codified in the passport system (in which Seto nationality did not exist). The language of instruction in schools was Estonian, and so all Setos in the region had the possibility of receiving an education in Estonian and sometimes prefer the ethnonym ‘Estonians’ to refer to themselves. 

The youngest informant was born in 1980 and the oldest in 1916. Mean age was 68 for Russian participants and 60 for Seto participants. The interviews were conducted with 25 Setos and 31 Russians. We spoke to 38 female and 18 male participants (Table 2). 

Although we looked for local participants, some of our interviewees revealed only at the end of the interview that they were born in another location. In all such cases, the period of residence in Pechorsky District was more than 20 years (which is the reason they considered themselves locals). Novozhilov [42] describes that while “part of autochthonous Russian population left this region, another part has dissolved into the stream of Russian migrants, whose number increased from 1990 to 2000”. As our aim was to explore plant use by the autochthonous population, we did not include these newcomers in the main sample and general analysis, as the size of the group is not comparable. However, we will discuss the attitudes and possible influences of the newcomers on the local population, on the basis of the twelve people (four men, eight women, mean age 68 years) we interviewed, in a separate section of the Discussion. 

We started our conversations with free-listing and then continued as semi-structured interviews. At the end of the interviews, we asked the informants for permission to take a picture or some dried specimens from their winter stores. We asked informants to describe which wild plants they use in various dishes, such as soups, pies, salads, condiments, smoked meats, desserts, and infusions. We specifically inquired about the use of tree sap. We also asked the informants to describe the wild food plants, they, or their family members, used during their childhood. Whenever possible, we also conducted participant observation at religious and other celebrations and requested to take field walks with the informants to collect voucher specimens. Informed consent was obtained at all times and the guidelines prescribed by the ISE Code of Ethics were strictly followed [43]. 

Informant interviews were anonymized, transcribed, and organized into an Excel spreadsheet in order to obtain detailed use reports (DUR) sensu [44], where the use of each taxon is distributed across ethnic groups and time of use in the life of an informant. Seto, Estonian and Russian phytonyms were recorded and used to qualitatively evaluate the level of phytonymical knowledge of the local population. Romanization of the Cyrillic script of the original Russian words was done according to the ALA-LC (American Library Association—Library of Congress) Romanization without Diacritics set of standards (https://www.convertcyrillic.com/#/).

The following time categories were identified according to the interviews:

PAST

long ago—during the generation of their grandparents or earlier;

abandoned in childhood—actively used in childhood (as it was considered a children’s food or was imposed or requested by the family);

abandoned in adulthood—practiced since childhood and abandoned during adulthood;

abandoned recently—practiced since childhood and abandoned within the last few years;

CURRENT

adulthood—started in adulthood and practiced since then;

only now—acquired within the last five years;

all time—learned within family and practiced since then.

For cross-cultural comparison, we calculated the Informant Consensus Factor (IFC) for use categories following the formula utilized in [45,46]:

$$IFC=\;\frac{N_{ur}-N_{t}}{N_{ur}-1},$$

where *N_ur_* is the number of uses in a use category and *N_t_* is the number of taxa in the corresponding category.

We included in the sample not only wild plants in the strict sense, but also some semi-cultivated taxa that easily run wild, such as *Armoracia rusticana*, *Amelanchier* spp., and *Rumex acetosa*, or that are cultivated for non-food purposes, like *Caragana arborescens*. 

The data were visualized using RAW Graphs [47] and the Venn diagrams tool published by the laboratory of Bioinformatics & Evolutionary Genomics lab at the University of Ghent (http://bioinformatics.psb.ugent.be/webtools/Venn/ (accessed on 12 May 2019)). 

The botanical nomenclature was checked against The Plant List [48], while the botanical family information was additionally verified against the Angiosperm Phylogeny Website [49]. Voucher specimens collected during fieldwork were deposited at the Komarov Botanical Institute of the Russian Academy of Sciences (Saint Petersburg, Russia) under the following numbers: LE 01063392-461, LE 01063463, LE 01063465, LE 01063466, LE 01063469, LE 01063477, LE 01063496, LE 01063498, LE 01063504-6, LE 01063510-14, LE 01063544, LE 01063578, and LE 01063946 (http://en.herbariumle.ru/ (accessed on 30 June 2020)). 

### 2.7. Cross-Border Comparison

We compared the data of the present study to that coming from a similar study [13] carried out simultaneously, and according to the same methodology, in Estonian settlements inhabited by Setos and Southern Estonians (Võro). The past and current wild plant uses in Russian Seto (25 people) and Estonian Seto (37 people) samples as well as local Russian (38 people) and Estonian (35 people) groups were compared. To conduct cross-border comparisons, we used the Jaccard Similarity Index (JI), following the methodology used in [50]:

$$JI=\left[\frac{C}{A+B-C}\right]\times 100,$$

where *A* is the number of wild plant taxa reported in sample *A* and *B* is the number of wild plant taxa reported in sample *B*, and *C* is the number of taxa common to both samples. We compared two sets of data: (1) ethnospecies recorded three or more times to compare the "core" sets, and (2) all recorded ethnospecies to compare ways of experimentation in the observed communities.

For the calculation of JI we considered as one taxon the following species, according to their emic categorization: *Alnus* spp., *Betula* spp., *Mentha* spp., and *Rosa* spp. At the same time, we regarded *Trifolium* spp. (including *T. pratense* and *T. medium*) and *Trifolium montanum* as two taxa, as the latter was referred to by different names or descriptions by the study participants. *Armoracia rusticana* was not included in the Estonian sample as it is cultivated with great care due to the peculiarities of the habitat, but it was kept in the Russian sample as it often escapes from gardens, tends to naturalize more easily, and is generally treated as a weed. Non-essential use of cultivated plants (mainly the leaves of *Ribes nigrum*) was also included in the sample, see [51].

## 3. Results and Discussion

### 3.1. Limitations of the Study

Before describing the study results, we would like to address some limitations of our study. Since our sample is relatively small, the statistic curation of the obtained data does not provide consistent results for the number of wild plants used in different use categories. We have performed Wilcoxon test and Kruskal–Wallis test that were not sensitive enough and therefore provided opposite results for the compared groups (see Table 3). While for Wilcoxon test, the *p*-value has by far exceeded 0.05 in all groups, meaning that null hypothesis about the group similarity is confirmed. Kruskal–Wallis test reveals that the p-value is significant for all groups except the pairs of Estonians/Russian Seto and Estonians/Russians, thus proving the opposite for almost all groups.

### 3.2. Current, Past and Historical wild Food Plant Uses

#### 3.2.1. Current Uses

We recorded 1388 detailed food uses of 72 taxa in 31 families: 60 taxa were identified on the species level, 10 on genus level, and one on family level; one ethnotaxa was not identified (Table 4). The most popular families were Rosaceae (15 taxa), Lamiaceae, and Asteraceae (10 taxa in each). The food uses were distributed across 22 emic use categories that included snacks, soups, soaked berries, jam (boiled and raw), pie filling, drinks made from berries (kissel or mors), drinks made from tree sap (fresh or fermented), infusions (tea and coffee substitutes), frozen preserves, additives for food in general and for lacto-fermentation, salads, mousse, and children’s snacks. The most common food categories were tea substitutes (290 DUR), jam (205 DUR), and snacks (186 DUR). 

The most frequently reported plants were the berries of *Vaccinium vitis-idaea* (142 DUR), *Vaccinium myrtillus* (141 DUR), and *Vaccinium oxycoccos* (140 DUR), the acidic leaves of *Rumex acetosa* (75 DUR), the fragrant seeds of *Carum carvi* (77 DUR), and the sap of *Betula* spp. (82 DUR).

**Figure 2:**
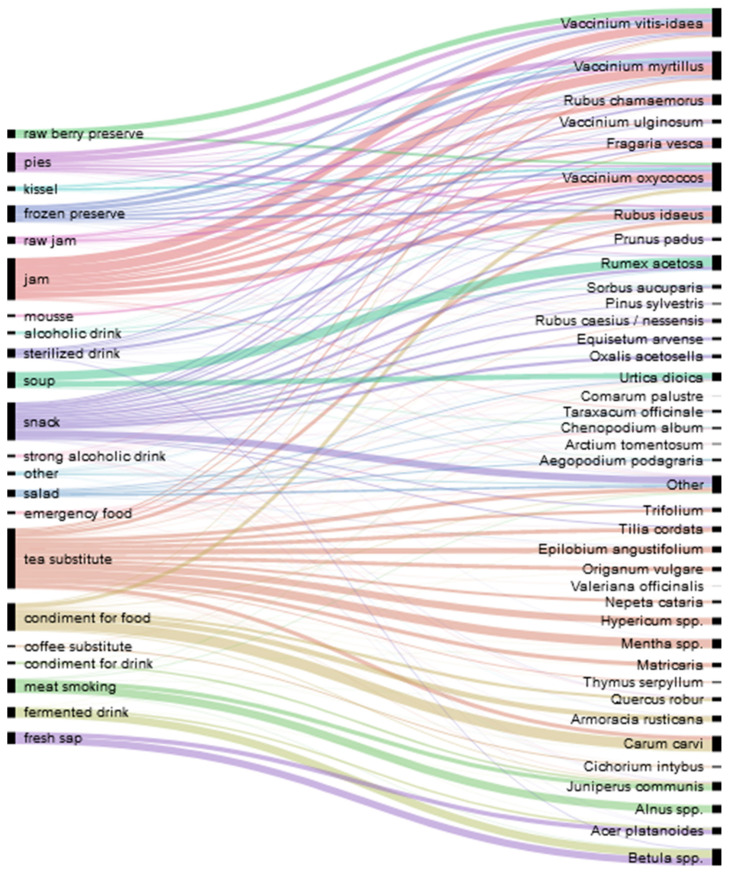
Wild plant taxa used by Setos and Russians as well as dishes made from them.

Figure 2 illustrates the distribution of different emic food categories among locally collected wild plants. The most popular food categories are jams that are primarily made from various *Vaccinium* berries and teas made from fragrant herbs such as *Mentha* spp., *Hypericum* spp., *Trifolium* spp., *Origanum vulgare*, the flowers of *Tilia cordata*, the seeds of *Carum carvi*, and some others. Wild plants are used as condiments in winter preserves: *Carum carvi* seeds and *Vaccinium oxycoccos* fruits go with fermented cabbage, and *Ribes nigrum* leaves, *Quercus robur* leaves, and *Carum carvi* seeds are put into fermented cucumbers.

**Figure 3:**
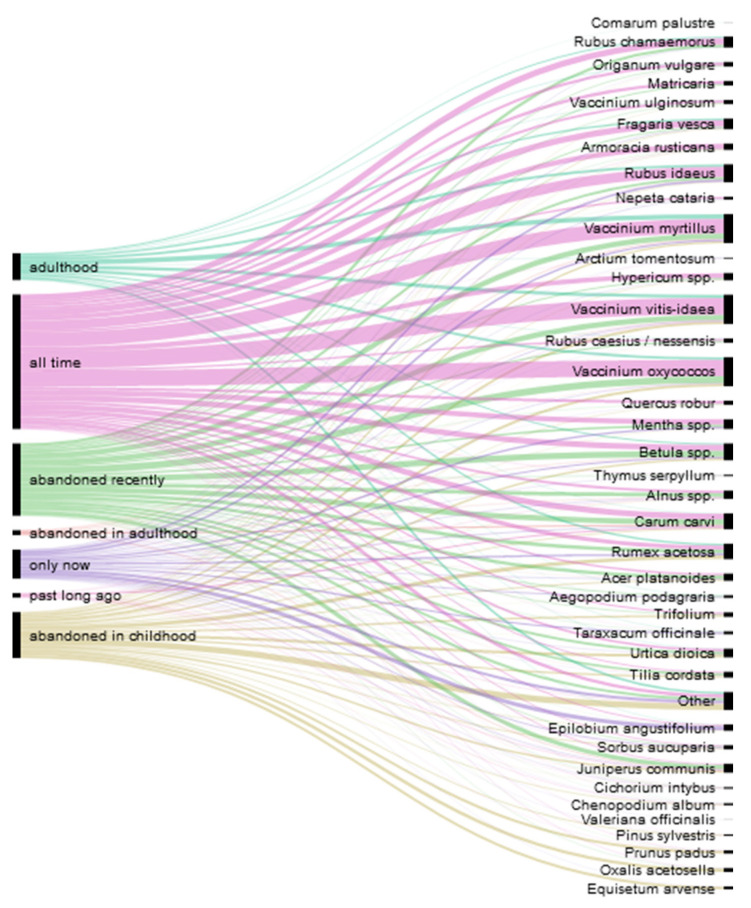
Diachronic distribution of wild plant use over the lifetime of the study participants.

The snacks category mainly represents childhood snacks as we specifically asked about the plants that were consumed or tried during youth but no longer eaten (see also Figure 3). Therefore, this category features all forest berries, acidic plants including *Rumex acetosa* and *Oxalis acetocella*, the berries of *Prunus padus* and the frostbitten berries of *Sorbus aucuparia*, and the strobilus of *Equisetum arvense* (Russian ‘pupyshi’). Spring shoots, needles, and the cambium of *Pinus sylvestris* were mainly reported by Seto participants. A couple of Russian participants mentioned eating the white sugary stalk bottoms of *Schoenoplectus lacustris (L.)* Palla (Russian ‘trosta, kamysh’). A general preference for sugary snacks was characteristic of those who grew up during wartime or shortly thereafter.

“Well, it’s like […] a little stem, the size of a finger. There’s a little cone, one did not eat it. And this stem, it was sweet. I don’t know why but we sucked it. We would suck it and spit it out. We wanted something sweet; there were no sweets then”.

(F, Russian, b. 1941)

Children used to suck on the sweetish stalks of *Equisetum arvense* as described in the above interview. Otherwise, they would suck nectar from the flowers of *Trifolium pratense* and *Primula veris*, or the sweet juice from the stalks of various Poaceae species (Russian ‘trava’, grass). One person remembered eating *Claviceps purpurea* growing on rye during her childhood in the post-war years:

“You know, the post-war famine was terrible. Well, I didn’t suffer from hunger as such … For some reason we filled our stomachs with kiselka [*Rumex acetosa*], with barkanniki, and horsetails [strobilus-bearing stems of *Equisetum arvense*]. In rye, they say it is poisonous; we ate ergot [*Claviceps* *purpurea*]. These little black horns. Now they treat rye, and it [*Claviceps* *purpurea*] doesn’t grow. It is considered […] and we in childhood, when rye was abundant [ate] these little black horns. Well, ergot, now I know the name. We ate ergot and no one was poisoned”.

(F, Russian, b. 1945)

#### 3.2.2. Past Uses

**Figure 4:**
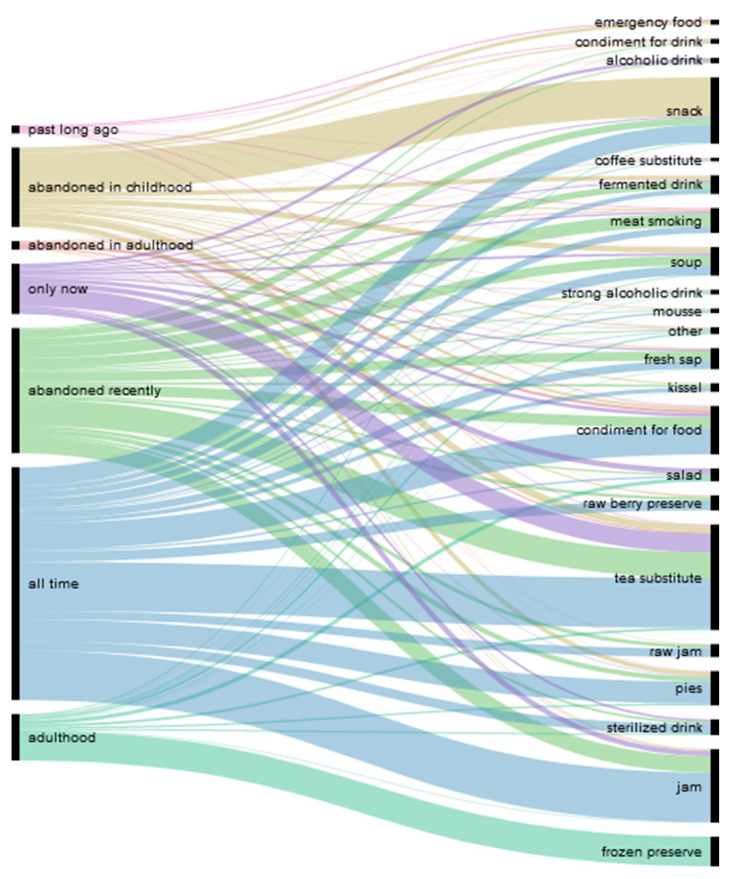
Diachronic distribution of the dishes prepared over the lifetime of the study participants.

Figure 3 and Figure 4 demonstrate the temporal changes in wild plant use. The majority of food uses were preserved over time. It seems that while the set of consumed species remains intact, preparation methods might change. This process is caused by infrastructural, economic, and cultural developments. One of the main tasks in the yearly cycle was to preserve the yield and not let it perish. The ways of preserving wild foods have changed over time. For example, forest berries were preserved without sugar due to its scarcity in the early post-war years. However, due to the risk of over-fermentation and mold, no one was able to predict how much should be preserved. Later, the availability of sugar allowed for the making of sugary jams (Russian ‘varen’e’). Some berries were simply crushed with sugar, without thermal processing (raw jam), using widely available manual mincing machines (our participants called it in Russian ‘perekrutit’s sakharom’, mince with sugar). The jams were stored in the basement and later in the refrigerator when villages were connected to the electricity grid. As home freezers became available, the freezing of berries also gained popularity. Raw jams and the freezing of fruits that were acquired in adulthood have become quite widespread, but they have not completely replaced traditional jams.

“Yes, I freeze it. No, sometimes I make a bit of jam simply […] generally I freeze the bilberry”.

(F, Seto, b. 1950s)

The existence of numerous preservation techniques allows people to choose, for each type of berry, that which best fits their taste or health condition, in the case that they should limit sugar consumption. Below is a quite characteristic monologue about the different ways in which berries are consumed in a family:

“[Wild strawberries are] better eaten fresh. They add lemon, they say they don’t become bitter then, but we freeze them down, you crush them with sugar or simply sprinkle some sugar. And raspberries, too, become so delicious and tasty [this way]. […] Best of all are frozen raspberries and cloudberries because you take them out in winter—and they taste just like summer. Bilberries, well there’re not to everyone’s taste, we are not into them but we also freeze them”.

(F, Russian, b. 1955)

Among the reasons for the recent abandonment of multiple wild food plants, interviewees mentioned the lack of time due to working in the field and weak health, which does not permit long walks or crouching over the berries. Some participants mentioned the disappearance of *Thymus serpyllum* and many complained about the loss of *Carum carvi* due to the abandonment of pastures and fields and the subsequent lack of mowing. Some interviewees were able to purchase the most important berries in the market or via their social networks, while others were happy to be regularly supplied by their grown-up children. Some wild plants disappeared from use because people started to cultivate them for food (such as *Rumex acetosa*) or use their cultivated relatives for making tea (like various *Mentha* spp. and *Nepeta cataria*).

One recently abandoned wild food plant is *Betula* spp., and specifically the use of its sap. Study participants mentioned harvesting *Betula* spp. from late Soviet times to until recently. They drank it fresh and also fermented it to preserve it until the summer work in the field, when it was consumed in large quantities. Sometimes raisins were added to start the fermentation process. Refreshing fermented sap is also one of the favored drinks after a steam bath, on par with soaked berries, morses, kompots, and herbal infusions. The generation of the interviewees’ parents also used sap as a base for kvass. In this case, special loaves made from barley flour were baked until burnt and then fermented in the sap. In this preparation method, juniper twigs were put in the bottom of the barrel in order to filter out the saturated loaves while pouring the drink from a tap installed in the barrel. Juniper was also used, until the late Soviet era, to sanitize and deodorize all sorts of barrels that were used for fermented or salted winter stocks. For this purpose, a specially selected stone was heated and put in a barrel together with juniper twigs, and then hot water was added. Later, with the appearance of nuclear families, the volume of preserves decreased, barrel makers gradually disappeared, and barrels were replaced by glass jars. In the 1970s and 1980s, with the spread of preservation methods, the technology for sterilized birch sap appeared, and some lemon or orange started to be added, which reached convenience stores at that time. The recipes are still remembered by many study participants. Currently, birch sap is most frequently consumed fresh, and the remains are fermented. Much more rarely, the sap of *Acer platanoides* is harvested. Its taste is often described as sweeter than that of birch, but at the same time it is harder to catch it at the right time as it comes earlier in the year, and fermentation makes the maple sap slimy (Rus. ‘soplivyi’) and unpleasant for consumption. One interviewee reported drinking the sap of *Tilia cordata*. Historically, such use was also found in Estonia [56].

#### 3.2.3. Historical Uses

The ethnographic descriptions of the traditional food of Pskov region is quite scarce and even those describing the food of peasants very rarely mention any applications of wild plants. At the same time, the use of berries and mushrooms was considered quite common because the recipes using them do not contain any further clarifications or directions on how to find them. Moreover, wild berries were often sold by peasants in the market. This occupation was considered appropriate for young girls who had to earn their marriage dowry [57]. Women who sold berries or mushrooms kept the money they earned for their own use [58].

In 1803, during his travels around the western regions of Russia, which included Pskov, Russian chemist and geologist Vasilii Severgin recorded two recipes with wild berries. He highlighted a recipe for vinegar made from *Vaccinium oxycoccos*, and a recipe for *pastila*, a reduction from fruit juice, indicating that it comes best from *Ribes uva-crispa* and *Sorbus aucuparia* [59].

Starting from the second half of the 19th century, due to the development of zemstva, local government units, peasant life began to be described by medical doctors who aimed to attract attention to the terrible living conditions and social problems leading to poor health [60,61,62]. Those descriptions agreed that the local diet was predominantly vegetarian as meat was allowed only on non-fasting days, which consisted of 244 days a year. The consumed vegetables were cultivated, the main crops being cereals (rye, barley, wheat, oats, and buckwheat), cabbage, potatoes, onions, beans, peas, and root vegetables (*Brassica rapa, B. napus*, beetroots, and carrots). The main dishes were bread, shchi, and kvass. Academic observers indicated not only the poor quality of the bread (see below), but also regular bread shortages that occurred almost every year just before the new harvest.

Among the rare descriptions of wild foods, there are bread additives that were used when flour became scarce in the summer.

“The main nutrition after rye, here, constitutes potato and cabbage; and also beetroots, cucumbers, radish and others. […] In the case of need, peasants use *pushnina* or *pushnoi* bread, i.e., not winnowed, with weeds remaining—*koster* and *metla* [most likely rye shives and *Agrostis* spp.], sometimes they add ‘lebeda’ (very rarely) [most likely *Chenopodium alba* [Here and further the conclusions are based on Pskov Oblast Dictionary with Historical Data [55] and Botanical Dictionary by Annenkov [63]]; recently in our area they have borrowed from Latvians the habit of adding to rye flour (in leavened dough) a dilution of ground potato (only in the case of need as well); they do not consume bark and leaves here”.

[58]

The root of *Alisma plantago* (local name *bobovnik*, common Russian name *vodianoi podorozhnik*) was also reported as a flour additive that served to make flatbread during the yearly shortage of bread [60]. The Pskov Oblast Dictionary lists *Caltha palustris*, whose roots were also made into flour, under the name of *bobovnik* [55]. This is, however, most likely a misidentification of the local name, as the description of the plant in the text does not correspond to *C. palustris* and the plant itself is toxic unless boiled.

As the cuisine of Pskov region did not form a local brand like, for example, Karelian cuisine [64], the local culinary tradition was not described until the collapse of the Soviet Union. In 1991, the first book on Pskov and Novgorod culinary traditions was published [65]. It criticized the plain and uniform Soviet cuisine that did not take into account regional traditions and proposed relying on them in order to balance the problems otherwise caused by the planned economy. Curiously, the authors quoted a famous observation of exiled Russian chemist Alexander Engelhardt (1832–1893) regarding the diet of peasants who chose their meals according to the perceived caloric capacity that would be needed to perform manual labor. According to his description, more dense foods were chosen for more physically taxing jobs and less caloric ones for less demanding tasks, cf. [66]. The recipes published in the book featured the most common forest berries and mushrooms. A special section was dedicated to the menu of Pskov-Pechory monastery that inferred the use of mushrooms and potentially berries in jams, but it did not provide any details of preparation or particular plant names. Precise instructions on wild plant use were present only in the section that emphasized their beneficial properties, which contained recipes that did not match either our field experience or the ethnographic data: for example, juice from nettles, carrots and lemons, or a salad made from the leaves of *Arctium lappa* mixed with onion and horse radish. The list of recommended wild species included salads and dressings, snacks, and infusions made from: *Urtica dioica* (salad with *Allium ursinum* and *Ficaria verna* Huds., also juice and nettle powder), *Arctium lappa* (also a preserve from the leaves), *Taraxacum officinale* (salad from the leaves, coffee substitute, and candy from the roots), *Plantago major*, and *Aegopodium podagraria*. The nature of these recipes and other similar examples [67] suggest that these recipes did not actually belong to the local tradition but were suggested by the authors on the wave of popularization of wild plant consumption that started in the late Soviet era.

Another, more recent book on Pskov culinary traditions is a collection of recipes of various traditional meals reported by the local inhabitants [68]. Twenty-five out of 110 recipes contain wild food plant ingredients: *Chenopodium album*, *Rumex acetosa*, and *Urtica dioica* in soups; *Carum carvi* as a condiment in breads (Russian ‘komy’), curd cheese (Seto ‘sõir’) and fermented cabbage; forest berries in desserts and tarts; *Vaccinium oxycoccos* in fermented cabbage and morses; *Vaccinium vitis-idaea* in soaked apples (both fruit and leaves); *Rubus idaeus* in desserts and drinks; *Humulus lupulus* in soft drinks and low alcoholic beverages; *Juniperus communis* in beer; *Menta* spp. and *Tilia cordata* flowers in infusions; and the sap of *Betula* spp. and *Acer platanoides* in fermented drinks (Russian ‘ber’ka’). Two recipes presented uses not confirmed in the field. The first one lists the leaves and cambium of *Tilia cordata* as ingredients in flatbread (Kun’ia settlement, south of Pskov Oblast). The second one, Rus.‘gorokhovye babashki’, is dated to the 1930s and features the seeds of *Vicia sativa* (Rus. ‘vika’) as the main ingredient of bitter but dense pancakes made during times of hardship.

### 3.3. Cross-Cultural Comparison

**Figure 5:**
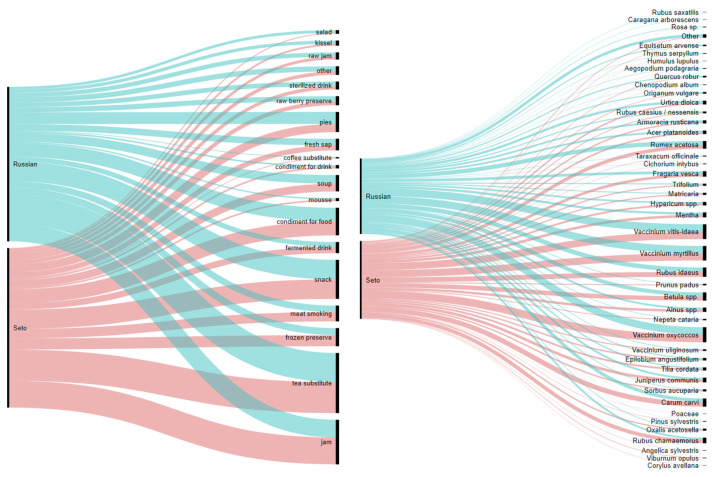
Cross-cultural comparison of food categories and wild plant taxa.

Cross-cultural comparison reveals a high level of homogeneity in the two studied groups with a core of 39 taxa that is the same for both of them (Figure 5). The Seto group has a higher intensity of wild plant use: 27 uses on average, in contrast to 21 for the Russian group. Moreover, Setos have a higher average number of plant taxa mentioned per user at 27.59, while for Russians the average number is 22.08. Both local groups, Setos and Russians, frequently reported drinks and preserves made from the forest berries *Vaccinium myrtillus*, *V. oxycoccos,* and *V. vitis-idaea*, birch sap, condiments, and tea from *Carum carvi*, and soup from *Rumex acetosa* made in the spring. The highest IFC for the Seto and Russian groups (>0.8) are for additives to lacto-fermented cabbage, cucumbers and mushrooms, tree sap drinks, jam, meat smoking, soaked berries, sõir, and soup. In the Russian group, a high rate was also found for mors and tea substitutes. The Seto group showed a high proportion of frozen preserves.

The use of fresh wild greens such as *Chenopodium, Aegopodium podagraria,* and *Taraxacum officinale* in salads is not characteristic of the local population. As one woman put it, "We are not grazers" (F, Russian, b. 1942), implying that "we", or her family at large, do not have a habit of incorporating wild greens into their diet, in contrast to those who do so, usually guided by popular literature on wild plants and also usually having come from elsewhere. While being aware of their potential as edible plants, she chose to adhere to the traditional list of wild taxa known since childhood.

The region avoided the main famines of the USSR, though it experienced some shortages in supply after WWII. The last famine occurred in 1862–1865 due to the potato blight epidemic that originated in the Baltic region. However, due to the unpredictable weather and relatively short vegetation period from May to September, famine bread recipes were widely in use at the end of the 19th century [58]. These techniques were remembered and used again during the supply shortages that occurred during and after WWII. Currently, the plants that are associated with times of hunger are not incorporated into everyday practices. In the local perception, wild greens are usually associated with the abstract hardships of war and post-war times. When we asked informants about wild plants added to salads and then, after receiving a negative response, gave the examples of *Urtica dioica* and *Chenopodium album*, they usually referred us to post-war bread recipes with *Chenopodium*, etc., stating that currently they do not consume such plants. For example, a Russian man born in 1939 said that they used to add *Chenopodium* to bread dough, make flatbread with *Urtica dioica*, and collect "white moss" in the forest, because the village authorities could only provide 2 kg of flour for a family for a month.

Daughter (Russian father, Seto mother, b. 1968): “I remember she [Russian grandmother] said that they used to eat goosefoot when there was a famine, goosefoot in post-war years or something. I don’t know what was used afterwards. They added it. So…”

Interviewer: “Did they eat raw goosefoot or add it to something?”

Daughter: “I think, they added it to the bread, when baking, for sure I…”

Mother (Seto, b. 1945): “No, we didn’t have that.”

Daughter: “Well, my grandma said.”

Mother: “But it is a Russian grandma.”

#### *Carum Carvi* and Ethnic Stereotypes

In one of the interviews, we shared an interesting exchange about foods with *Carum carvi* seeds acting as a marker of ethnic identity:

Interviewer: “You mentioned caraway. We thought that it was more of a Seto tradition… What do you consider yourself?”

Participant (F, Russian father, Seto mother, b. 1955): “You know, inside me it is even, just like I consist of two halves, it lives inside me in two halves. So one day I do feel Russian, but at times absolutely, I absolutely have a craving for… in any case, my food choices are Seto ones because I like this cuisine, like this simplicity. I do like caraway; in curd cheeses, bread, sweets, everything.”

Interviewer: “Sweets??”

Participant: “Yes.”

Interviewer: “Do you make them yourself?”

Participant: “No, I don’t. Formerly in Estonia they made chocolate bars with caraway; very tasty. By the way, I can’t find them nowadays for some reason. Maybe they exist…shortbread with caraway. Well, I like caraway in every form; in every food.”

Other Seto informants also linked the use of *Carum carvi* with such food categories as tea substitutes and sõir (Seto homemade curd cheese made with the addition of eggs and *C. carvi* seeds). At the same time, a Russian woman born in 1927, who spoke in very prominent Pskov accent, attributed *Carum carvi* to ‘gun’ba’ [55] and reported that her mother used to make curd cheese (Rus. ‘varila syr’), adding the seeds. Two more Russian people, born in 1952 and in 1960, also mentioned this plant name. It was used as a bread additive, seasoning for sauerkraut, and as an ingredient of ‘syr’/sõir.

Overall, we recorded 29 DUR for *Carum carvi* for the Russian group and 43 DUR for the Seto group. While sõir with *Carum carvi* seeds is usually described as a staple of Seto food [69], we encountered nine such uses of *Carum carvi* in the Russian group compared to 11 cases among Setos. None of our Russian interviewees declared a Seto origin of their parents or using the Seto language in the family, nor did they report borrowing this tradition.

*Carum carvi* is also used in sauerkraut (7 Russian/7 Seto DUR) and as a seasoning in other lacto-fermented preserves (1/2), as a tea substitute (7/12), and as a universal additive including bread seasoning (6/10). In the Russian group, there were 14 current, and 15 past uses of *C. carvi*, while in the Seto group there were 15 and 28 uses, respectively.

This finding, as well as the results of the following cross-border analysis, seems to support the interpretation of the ethnic situation in the region by Novozhilov [42], in which attachment to a certain locality can be as important as ethnic identity. See also the data from the cross-border comparison that shows a higher level of cohesion between Setos and dominant Russian and Estonian groups than between Setos across the border.

### 3.4. Cross-Border Comparison

Table 5 demonstrates that for widespread uses (at least three people) the greatest homogeneity of used plants (JI from 80 to 91) is among Russians and Setos living on the Russian side of the border, while the homogeneity of use between Estonian Setos and Estonians is slightly lower (from 73 to 89). For all taxa, however, the highest overlap is between Estonians and Estonian Setos (from 68 to 85), followed by Russians and Setos on the Russian side of the border (from 61 to 69). JI value between Seto groups across the border remains approximately 60 (57–66) in both cases.

The lower similarity indices for all taxa reflect the high level of experimentation and individualization of uses. To some extent this occurred during the Soviet era, yet some experimentation is reflected in the recently acquired uses. Also, we can observe high individual variation in the children’s snacks reported by our interviewees and those in Estonia. If we take into account only widespread past uses, the JI remains the same for Russian Setos when compared with both EE Setos and Estonians—this shows that despite the administrative border, RU Setos retained a strong connection with the Estonian side and lived in the Estonian cultural space. As our interviewees still remember the time when there was only an administrative border between the two groups of Setos and cross-border relations were much stronger, there is still a higher level of overlap for current uses between the two Seto communities compared to the overlap with the cross-border dominant groups. We can observe very similar, although even stronger, cross-border differences between members of the same ethnic group in the historical region of Bukovina, which is currently divided between Romania and Ukraine [11]. The relatively high similarity between Setos living in Russia and Estonians for all taxa continually used can be explained by the education provided in Estonian and the spread of Estonian-language media.

**Figure 6:**
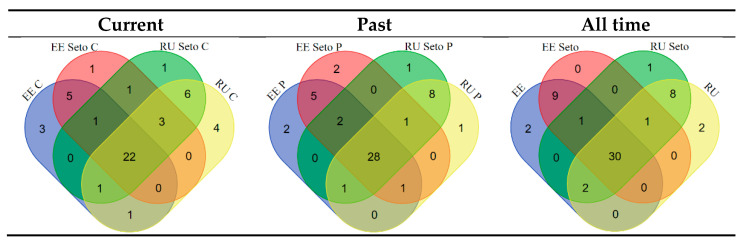
Overlapping of taxa used by Estonian (EE) and Russian (RU) Setos in the past (P) and currently (C). Only plants mentioned three or more times are analyzed.

Nevertheless, the level of cohesion across the communities is quite high. Indeed, there are few plants that are unique for each group, and those mentioned by more than three people are even fewer. Out of 56 ethnotaxa mentioned as currently used by at least three people, 30 taxa are used in all four groups (Figure 6). It is important to add that the absolute number of currently used taxa is shrinking, and the present differences are mostly due to the unequal abandonment of plants in the studied communities.

As shown in the diagrams, the majority of plants are used ubiquitously in the region, although there are particular plants specific to each group and two clusters of plants specific to Estonians and Estonian Setos on the one side and Russians and Russian Setos on the other side. These differences can be subdivided into two general cases:

(1a) Plants used in similar ways by different groups at different times—formerly used throughout the whole region but gradually abandoned: *Angelica sylvestris*, *Corylus avellana*, *Trifolium* spp., *Lamium album*, *Rubus saxatilis*, *Amelanchier spicata*, and *Humulus lupulus.* For *Viburnum opulus* and *Chenopodium album* we recorded their current use in Russia and archival use in Estonia. The stems of various Poaceae taxa are used as snacks across the border, and in Estonia this is namely *Phleum* spp. *Schoenoplectus lacustris* from Cyperaceae is also used as a snack in Estonia.

(1b) Plants that were acquired (and often abandoned) due to recent popularization—for example, the use of *Taraxacum officinale* in jams and salads.

(1c) Plants that are used for different functions on the two sides of the border.

*Origanum vulgare*—while in Estonia it serves as an additive to blood sausage, in Russia it is solely used in infusions. *Achillea millefolium* is used as a tea in Estonia and by Russian Setos and as medicinal plant in Russia. While in Estonia the tubers of *Equisetum arvense* are used as a snack, in Russia strobilus-bearing stems are used for this purpose.

(2) Plants that are unique to ethnolocal groups across the border.

We recorded exclusively in Estonia the use of cambium of various trees, usually as children’s snacks: most frequently *Pinus sylvestris* (16 DUR), but also *Betula* spp. and *Salix* spp. The resin and shoots of *Picea abies* are also used predominantly by Estonians.

*Thymus serpyllum* is used in tea by all groups except Estonians.

The use of wild *Ribes nigrum* leaves for the lacto-fermentation of cucumbers and mushrooms was recorded only in Russia. Wild *Cichorium intybus* is used as a coffee substitute in Russia, while in Estonia it was also used for this purpose, but only from cultivation.

There was also an important conceptual difference: in Estonia plants were used just for making tea, while on the Russian side they were used as tea substitutes. 

#### Plant Names

This section provides a comparison of Seto plant names recorded on both sides of the border. Interviews in Estonia and Russia were conducted in Estonian and Russian, respectively (except one interview in Estonian with a Seto informant who traveled from Russia to Estonia). Despite an obvious limitation—difference in efforts needed to switch between dialects of one language than between two different languages—given the lack of data on the Seto language, we draw a preliminary comparison of the available data.

Only a few participants on the Russian side of the border declared that they regularly speak Seto with their peers, and no one admitted to teaching Seto to their children. However, we encountered a willingness to teach at least some Seto to grandchildren residing in Estonia, especially given the recent publication of a Seto ABC-book [70]. The Estonian data also provides evidence of a language shift that started in the 1960s-1980s [12], thanks to the accessibility of professional education and careers that are only compatible with the use of the Estonian language. Nevertheless, Seto remained the home-spoken language, as the majority of the Seto interviewees in Estonia spontaneously answered in the Seto dialect and all claimed to speak the dialect at home. 

On the Russian side of the border, 16 of the 66 recorded taxa had recorded dialect names and for eight taxa we recorded previously undocumented names, which were elicited from 18 of the 25 participants (Table 6). Only dialect names were recorded for *Carum carvi*, *Rumex acetosa*, *Tilia cordata* and *Urtica dioica*. The most frequently given Seto and Estonian names were those for *Vaccinium oxycoccos*, *Vaccinium vitis-idaea*, *Rubus idaeus*, *Betula* spp., and *Juniperus communis*. Berries like *Vaccinium myrtillus*, *Rubus idaeus* and *Rubus chamamemorus* were referred to with more conventional Estonian names.

For comparison, on the Estonian side, 37 of the 62 recorded taxa were named by Setos in a dialect (Seto/Võro) and dialect plant names were encountered quite often. Out of the 647 occasions in which the name of a wild food plant was mentioned, 175 were dialect names, two Russian names (a recent introduction of Ivan-chai [71]), and the rest were names used all over Estonia, including the whole of South Estonia. Five taxa were referred to by their dialectal name on more than 10 occasions: *Vaccinium oxycoccos* (29), *Matricaria discoidea* and *Vaccinium vitis-idaea* (both 18 occasions), *Rubus nessensis* (14), and *Thymus serpyllum* (11). Only one person used a single dialectal name, while five or more dialectal names were used by 17 people, of whom one used 19 dialectal names.

### 3.5. Factors Affecting the Use of Wild Food Plants

#### 3.5.1. Collectivization and the Generation Gap

Many of our interviewees admitted that they were brought up by their grandparents, and more precisely by grandmothers, as their parents generally had little time while working on collective farms. Those who possessed a private plot of land (almost everyone in Pechorsky District) were also obliged to meet milk, egg, and other farm product quotas. The surplus could be sold in the market. As all the pasture land belonged to sovkhozes, or state-owned farms, they had to harvest hay from around ditches and in the forest.

“So they woke up in the morning, and they have their own cattle, right? Meanwhile the cattle and all this stuff, some of them you feed, some of them you bring to the pasture. Then work in the kolkhoz. Come home for lunch, it’s time to milk the cow. While going to milk the cow, chewing a piece of bread on the go—it’s already time to go back to the kolkhoz. Come back home at 7, the cows are back from the pasture, it’s time to care for them again. And then in the evening you have to find some hay for your own cattle… So it was, I’m thinking about it now, how they lived and how they survived, and when they… So they likely woke up at 3 am and went to bed at 12. […] They would wake up in the morning, run somewhere, to some ditches—it wasn’t allowed to mow anyone’s fields, they belonged to the kolkhoz. So, where there were some ditches, somehow they would mow some. Then they would come home, and go to work, and so it was. And then in the evening, the cows would come back, other cattle would get their fodder too, and they had to bring that hay in. It was a total nightmare”.

(F, Russian, b. 1942)

Some interviewees who lived a couple kilometers from the border remember going to Estonia in order to obtain some hay. As a result, their parents almost never had time to go to forest, and if some berries were collected, it was done while collecting brush wood or harvesting the peat used for heating. Therefore, it was mainly grandmothers who went to the forest with their grandchildren. Thanks to this, some information that was discarded by the informants’ parents’ generation was passed on to that of the interviewees who were growing up at the time of rising interest by the state in wild resources and new provisions, procured from the wild, passed on via local shops and sometimes schools (see below). The following dialogue between daughter and mother is quite characteristic:

Daughter (F, Seto, b. 1968): “[…] there is kislitsa [Russian ‘kislyi’—sour] growing in the woods. Heart-shaped leaf, it is rarely ripe and I remember my grandmother told me that you can pick it, it is sour. […]”

Mother (F, Seto, b. 1935): “We called it zaiach’ia kapusta [literally rabbit’s cabbage].”

Daughter: “It’s not zaiach’ia kapusta at all.”

The both names are used in the region to indicate *Oxalis acetocella*, and its Seto name jänesekapsas also means rabbit’s cabbage. The daughter, who was brought up by her Russian grandmother, had a much better idea of the wild plants collected in the forest and even gave a different name for *Oxalis acetosella* (‘kislitsa’) than did her mother (‘zaiach’ia kapusta’) as she learned it from her grandmother.

Biology school teachers and those of primary school grades could not complain about the ecological knowledge of their pupils. As one retired teacher said, the students “would tell you more [than the teacher], as they came from the village” (F, Seto, b. 1950s).

#### 3.5.2. Possible Literature and Media Influence

In general, the local population was quite reluctant to refer to any wild plant guides. The generation of our interviewees’ parents used to subscribe to magazines such as *Krestianka* (published between 1922 and 2015, 22 million copies sold as of 1990) and *Rabotnitsa* (published since 1914, 23 million copies sold as of 1990) though the majority of locals did not have either money or time for this:

Interviewer: “Did you use any recipes from culinary magazines, for example, *Rabotnitsa*, *Krestianka*?”

Interviewee A (F, Russian, b. 1962): “Which culinary magazines, dear!”

Interviewee B (M, Russian, b. 1964): “It’s expensive, mind you.”

Interviewee A: “No culinary magazines.”

Interviewer: “So you did not subscribe to any journals?”

Interviewee A: “No, my mother subscribed to *Krestianka* but I do not remember any culinary recipes, I don’t remember it.”

Interviewee B: “We had other things to worry about than fancy stuff; we had to bring in hay.”

Interviewee A: “We had to work from dawn till dusk… Which recipes? Put the cabbage to ferment, salt the cucumbers [for lacto-fermentation], boil the jam.”

**Figure 7:**
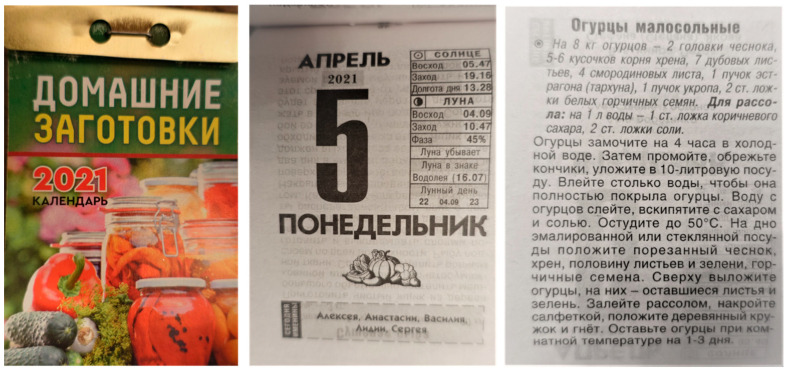
Contemporary tear-off calendar ‘Homemade preserves’ for 2021 bought in Pechory with a recipe of salted cucumbers with leaves of *Quercus robur*, *Ribes nigrum,* and *Artemisia dracunculus* on the reverse-side of a page indicating the date.

Some declared, however, that they used to save or store in scrapbooks some recipes from tear-off calendars that were popular in late Soviet times: “Of course, I do not agree with all of them, they tend to lie much more frequently these days. But earlier we somehow used [calendars] quite so often, it was somehow interesting, I used to buy them” (F, Russian, b. 1960). This was one of most widespread types of calendars in Soviet Russia up until the 1990s: each page of such calendars represented a day of the year and its reverse side contained tips for everyday life or some trivia. There were calendars dedicated to different subjects like winter preserves, household tips, etc. The information published there derived from lengthy household encyclopedias that were more expensive than, and not as handy as, a small calendar (Figure 7).

One Seto woman with higher education admitted to using some wild plants in salads but she clearly indicated that her knowledge came from written sources, including, for example, ‘honey’ from dandelions: “I made dandelion jam several times. But this was yet another thing, following magazines or books; goutweed, dandelions, and nettle” (F, Seto, b. 1955).

Currently, those who own a computer or a smartphone occasionally look for new recipes using search engines and social networking sites. However, the Internet is only used to find the details about already known information and not for discovering something new.

Interviewer: “You say you would rather look something up on the Internet?”

Interviewee (F, Seto mother, Russian father, b. 1968): “Yes, you’ll find it quicker.”

Interviewer: “A recipe for what? What did you look up last time?”

Interviewee: “The recipe for salt for fermenting cucumbers—for a liter of brine.”

Interviewer: “I see, different measures.”

Interviewee: “Yes, because usually there is a three-liter jar, and this time there was some leftover; and I had to use an unknown container. And you don’t know how much salt for a liter of water and not for three liters as usual”.

#### 3.5.3. State Procurement of Wild Resources

While collective farms regulated the interaction between farmers and cultivated plants, late Soviet policy introduced another structure that influenced the relationship of the local population with wild plants, the procurement office. This office was organized by the Tsentrosoyuz, Central Union of Consumer Societies, a commercial network aimed at the procurement and distribution of agricultural products that has its roots in late Imperial Russia and was eventually institutionalized in 1928. It procured and redistributed the produce of farmers locally, as well as on the state level, via a network of procurement offices (*zagotkontora* or *zagotpunkt*) and local shops (*raipo*—regional consumer society or *sel’po*—rural settlement consumer society).

Today, the system of *raipo* in the Pskov region is still active in the villages of Pskov Oblast, although now they have to compete with big commercial supply chains with regard to distribution and with individual entrepreneurs in terms of procurement.

According to our study participants, the procurement campaign for wild resources was always present but gained popularity in the 1980s. Procurement offices accepted wild forest resources from the local population: bark of *Salix* spp., fruits of *Vaccinium myrtillus*, *Vaccinium vitis-idaea*, *Vaccinium oxycoccos*, *Sorbus aucuparia*, and others. Some families collected berries that they did not usually utilize themselves, such as rowan.

Interviewer: “Did you use rowan?”

M, Seto, b. 1952: “Rowan, very rarely.”

F, Seto b. 1950: “Rowan, we picked them. And why did we pick them? Perhaps, we traded them. Somehow… they were accepted [at a procurement office]. And ourselves, not sure we made anything from them. But I remember that in childhood we climbed the trees and picked them.”

**Figure 8:**
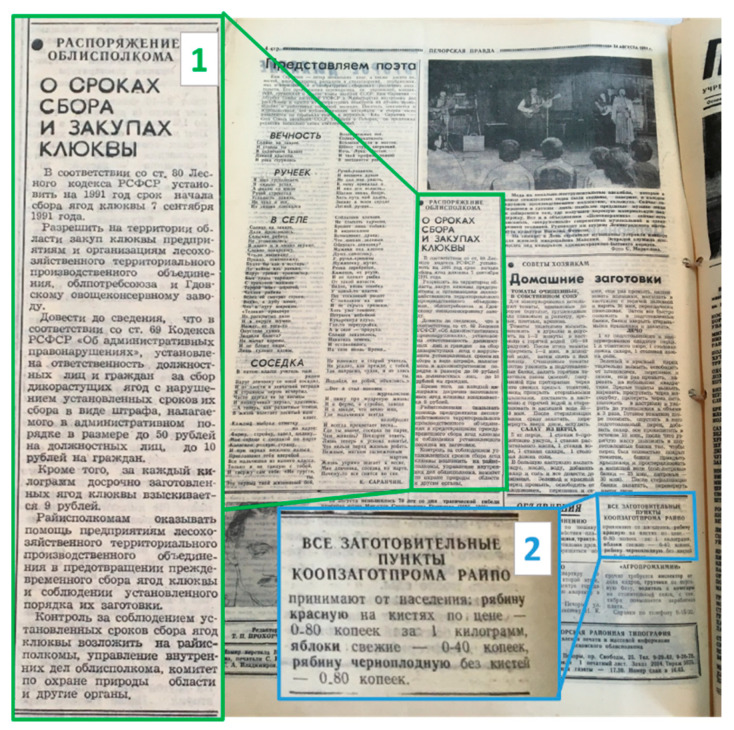
Last page of the local newspaper ‘Pechorskaia Pravda’ from 24 August 1991. 1. Decree of the district executive committee ‘On the dates of harvesting and procurement of cranberries’. 2. Announcement from the district procurement office (transformed into a cooperative) about accepting rowan berries at 0.8 rubles/kg, apples at 0.4 rub/kg and x *Sorbaronia mitschurinii* berries at 0.8 rub/kg.

Some participants mentioned that sometimes it was difficult to find berries in the forest due to high competition, which resulted in the harvesting of unripe berries, especially cranberries. Figure 8 demonstrates an order of a regional executive committee setting the start date of cranberry harvesting on 7 September, as well as fines for those who did not respect this date. A collector would be fined 10 rubles for early collection and 9 rubles for each kilogram of cranberries, and a procurement office was to pay 50 rubles if they did not respect the start date.

Due to the extremely low cost for raw materials, this task was considered suitable for children. However, later, new incentives were introduced to motivate the local population. Those who bought shares in the local *raipo* (i.e., a local office of the consumer society that operated the village convenience stores and procurement offices), could count on some scarce goods that were not accessible otherwise.

“There was a thing that you had to be a stakeholder. I remember; when I first had kids, it was nearly impossible to buy diapers for the babies; you had to have a certificate, I remember it as clear as day. You had to be a stakeholder; a member of the *raipo*. So, they were only sold to the members of the *raipo*”.

(F, Seto, b. 1960)

#### 3.5.4. Wild Plants as an Economic Resource

The borderland position of Pechory made it an important market location: a customs point was installed there as early as 1662, and the current check point is only 2 km away from the small historical center. Every weekend, the market is flooded with visitors from across the whole district. Cars with Estonian number plates can also be frequently seen. The stationary stands are attributed to the respective stand renters, confirmed by plates with their surnames, but the weekends are characterized by the abundance of mobile stands of local farmers who do not have time to go there every day. At the market, one can buy fish from Lake Pskov, honey, dairy products, local vegetables (potatoes, onions, carrots, beetroots, tomatoes, and cucumbers), and cultivated and wild berries depending on the season: blackcurrant, *Vaccinium mirtyllus*, *Fragaria vesca* in smaller quantities, *Vaccinium vitis idaea*, *Vaccinium oxycoccos*, *Viburnum opulus*, and *x Sorbaronia mitschurinii*. The most commonly sold mushrooms are *Boletus edulis*, *Leccinum* spp., and *Cantharellus sibarius*. Several stands also sell dried herbs and homemade preserves made from mushrooms and *Armoracia rusticana*.

During the Soviet era, despite the fact that individual trading was often in the grey area, the Pechory market was an important center of trade for the local community, including from the residents of Estonia. An inhabitant of Pechory describes the role of the local market as follows:

“Even our family had a goat, called Kat’ka. But she was so stubborn. My mother suffered and suffered with her, but then we brought her to the market; sold her. […] They say, after the war there was famine everywhere. We didn’t have it here. Here, we had individual farms, it was them. As soon as they were able to work here, you know, there was everything in the market. Milk, sour cream and butter, whatever you wanted”.

(M, Russian, b. 1955)

Not only was it possible to get rid of the excess of homemade products, many individuals also aimed to sell their produce at the market. The abovementioned interviewee later mentioned that his father used to grow vegetables, such as tomatoes and cucumbers, in the home garden within Pechory and that his father’s favorite pastime was selling them at the market.

**Figure 9:**
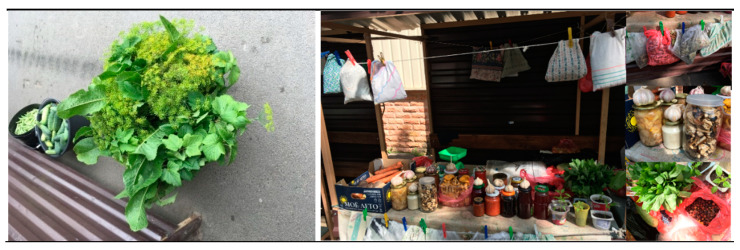
Pechory market. Left: Set of herbs for cucumber lacto-fermentation: *Armoracia rusticana* leaves, *Anethum graveolens* stalks with seeds, and *Ribes nigrum* leaves. July 2018. Right: homemade preserves made from mushrooms and wild and cultivated plants. August 2019. Photos by Olga Belichenko.

During numerous visits to the Pechory market, we observed only a couple of permanent stalls specializing in products made from wild and semi-wild plants, selling mostly fresh greens, dried herbs, or homemade preserves (see Figure 9). We identified the following categories: herbs for the lacto-fermentation of cucumbers; tea substitutes (for example, *Hypericum* spp., *Origanum vulgare*, and the fruits of *Rosa* spp.); fresh greens (*Rumex* spp.); fresh berries and preserves made from them; and fresh, fermented, or dried mushrooms. However, in addition to those stalls, we encountered different sellers, on different days, just at the entrance to the market, indicating that they did not have a permanent license; these sellers had brought forest berries and mushrooms that they collected themselves.

One of the current sellers (F. Russian, b. 1957) at the Pechory market with the widest choice of homemade preserves, admitted that she adapted her selection of products according to both requests from clients and information learned from fellow sellers at the market (see also [72]). For example, she remembered trying some *Tilia cordata* sap that was sold at the market and declared that she has recently tried to make ‘honey’ from *Taraxacum* flowers, thanks to the advice of colleagues.

#### 3.5.5. Immigration during Soviet and Post-Soviet Times

Unlike highly urbanized neighboring regions such as Leningrad, Estonia, and Latvia, Pskov Oblast, and more precisely Pechorsky District, remained an agricultural region. Nevertheless, it attracted a wave of incomers that is becoming more evident in the context of overall depopulation. Among our interviewees, we noted three groups:

(1) orthodox believers looking to reside near the only officially operating monastery in the USSR,
(2) military personnel and their families,
(3) ‘downshifters’(megapolis residents who came to Pechory in search of a quiet life).

The third group requires more attention as the influx of such downshifters has increased since 2000. Some of the villages that we visited were mostly populated by such immigrants. However, since then the nature of such immigration has changed. While for Soviet downshifters this was a personal choice, the current incomers tend to organize in communities for whom this choice is a life project (see more in [73,74,75].

Having grown up in an urban context, these groups tend to rely on popular guides to gardening and wild resources that became abundant in the era of procurement of wild resources. The wild food plant uses reported by this group of participants seemed inconsistent with the local tradition, but at the same time coincided with the recommendations found in popular literature (for example, [76]). A Russian woman (b. 1953) with higher education, who came from Leningrad, reported her favorite childhood book by Soviet botanist and wild food popularizer Nikolai Verzilin [77]. The following is the list of diverse plant uses reported by incomers to the study area: wine made from *Taraxacum* flowers; salad made from *Arctium tomentosum* leaves and its stems as a snack; *Taraxacum* and *Arctium* roots as coffee substitutes; salted preserves made from *Aegopodium*, *Taraxacum*, or *Urtica dioica* leaves; snacks and salads made from the leaves and young shoots of *Epilobium angustifolium*; *Plantago major* as a seasoning for fermented cucumbers or mushrooms; tea made from *Agrimonia eupatoria*; and dry powder seasoning for soup made from *Urtica dioica*.

Such practices are observed by the local population in small villages, but they are rarely borrowed or introduced into their own practices. During our research we were often directed by locals to such newcomers (though not described as newcomers) who were presented as being especially knowledgeable about wild plants, in contrast to the people we interviewed.

## 4. Conclusions

While wild plants do not constitute a substantial portion of the local diet, the repertoire of used wild plants is consistent. Almost all taxa are shared by both local communities. The list of plants used by Russian Setos is slightly narrower, yet the number of plant uses higher, than among Russians. Although some locals tend to recognize the use of particular plants like *Carum carvi* as ethnic markers, our data did not correspond to such a distinction.

Wild plants have historically been an economic resource for women and children in the region. The tradition of collecting them did not disappear during the decades of collectivization thanks to the cross-generational knowledge transfer from grandparents (usually grandmothers) to grandchildren. Later, local markets and state induced procurement in late Soviet times played their role in keeping the practice alive, although sometimes traders collected plants that they would not have used themselves. Since then, procurement points have been marginalized due to extremely low purchase prices. Currently, it is the local market in Pechory that plays an important role in the local knowledge transfer.

Interestingly, residents on the Russian side of the border seem to be united around the idea of the rejection of certain wild plants rather than actual use. Setos and Russians are reluctant to use plants associated with famine, such as *Chenopodium album*, *Urtica dioica* (except for soup), and *Aegopofium podagrarium*. However, other recipes, brought by newcomers, or borrowed from popular literature, like ‘honey’ from *Taraxacum* flowers, are sometimes incorporated into practice, mostly by people with higher education.

The cross-border comparison revealed that Seto groups are closer ethnobotanically to the dominant ethnic groups immediately surrounding them than they are to Setos across the border, especially where the most commonly used taxa are concerned. This confirms our assumption that for changes to appear a longstanding solid border is not necessary. Moreover, Setos in Russia seem to be ethnobotanically more Russified than Setos in Estonia are Estonified. The main reason for this, most likely, may be the larger number of Seto residents in Estonia and thus stronger Seto community. As the same tendencies can be seen on the level of plant names, we may conclude that those Setos who decided to remain on the Russian side of the border are highly assimilated with local Russians and have stronger connections, in part through family ties, to Russian than Estonian culture. From a theoretical standpoint, this shows that the evolution of wild food plant use also depends on the administrative choices in the region (such as local legislation, procurements, market regulations, education and other aspects decided on the local level).

Future studies need to pay closer attention to the specific role policies play in the evolution of local ecological knowledge of ethnic minorities, starting from a shift in marriage patterns to sociolinguistic factors such as the home language and the language of education and media.

## Figures and Tables

**Table 1:** Seto population of Pskov Oblast according to the estimation by Manakov [41].

Year	1959	1970	1979	1989	2002	2010
Estimation	4500	2360	1630	950	350	230
Census	-	-	-	-	172	123

**Table 2:** Demographic structure of the study participants.

Ethnic Group	F	M	Oldest	Youngest	Sample Size	Religion	Education
**Seto**	15	10	Born 1940	Born 1980	25	Orthodox	Vocational 22, higher 2, secondary 1
**Russian**	23	8	Born 1916	Born 1970	38	Orthodox or atheist	Evenly divided among secondary, vocational, and higher education

**Table 3:** *P*-values for Wilcoxon test (right top) and Kruskal–Wallis test (bottom left) for the compared ethnic groups.

	EE	EE Seto	RU Seto	RU
**EE**	X	0.8232	0.7977	0.4523
**EE Seto**	0.00638	X	0.6551	0.3335
**RU Seto**	0.2106	0.03156	X	0.5853
**RU**	0.1289	0.04577	0.01267	X

**Table 4:** Use of wild food plants by Setos and Russians today and in the past.

Latin Name	Local Name	Parts Used	Preparation	Food	Setos	Ru	Setos	Ru
					Past		Current	
**Adoxaceae**								
*Viburnum opulus* L. LE 01063405	RU Kalina	Fruit	Frozen and boiled	Jam	1			
			Frozen and dried	Tea			1	
			Frozen	Snack			1	
				Raw jam			1	
				Juice			1	
**Amaranthaceae**								
*Chenopodium album* L.	RU Lebeda	Young leaves	Baked	Bread additive	1	1		
			Boiled	Soup	1	1		
			Fresh	Salad				1
**Apiaceae**								
*Aegopodium podagraria* L. LE 01063450, LE 01063430	RU Snytʹ, lapka ^RD^	Leaves	Baked	Bread additive		1		
			Dried	Tea				1
			Fresh	Salad	1	1	1	1
*Angelica sylvestris* L.	RU Dudochki ^NF^, dudnik, morkovnik ^RD^	Stalks	Fresh	Snack	3	1		
*Carum carvi* L. dsPCH19-010	RU Tmin, gun’ba ^RD^; Kurinaia lapka ^NF^ S Küümne ^B^	Seeds	Fresh, dried	Seasoning for bread	4	2		2
				Seasoning for sõir	10	2	2	6
				Seasoning for fermented cabbage	4	6	3	1
				Seasoning for fermented cucumbers			1	1
				Seasoning for salted mushrooms			1	
				Other seasoning	2	2	4	
			Dried	Tea	8	3	4	4
**Asteraceae**								
*Achillea millefolium* L. LE 01063441, LE 01063544, dsPCH19-006, dsPCH19-004	RU Tysiachelistnik, tysiachelistvennik ^RD^	Leaves	Fresh	Snack		1		
		Aerial parts	Dried	Tea			1	
*Cichorium intybus* L.	RU Tsikorii	Roots	Dried and roasted	Coffee substitute	3	1		2
*Cota tinctoria* (L.) J.Gay LE 01063394	RU Zheltaia romashka ^RD^	Aerial parts	Dried	Tea			1	
*Tripleurospermum inodorum* (L.) Sch. Bip. (sometimes collected in the wild instead of *Matricaria recutita*) LE 01063445, LE 01063422, dsPCH19-012	RU Romashka S Kummel ^A^, teekummel ^A^	Aerial parts	Dried	Tea		1	1	2
*Matricaria discoidea* DC. LE 01063395, LE 01063444, LE 01063416, dsPCH19-011, dsPCH19-019	RU Romashka polzuchaia ^NF^, r. dvorovaia ^NF^, r.ulichnaia ^NF^ S Morohain ^NF^	Aerial parts	Dried	Tea	1	2	3	4
			Fresh	Seasoning for fermented cucumbers			1	
*Taraxacum officinale* Weber ex F.H. Wigg (Coll) LE 01063407	RU Oduvanchik	Flowers	Boiled	Jam		1	2	1
		Leaves	Fresh	Salad			2	1
				Snack		1		
*Tussilago farfara* L.LE 01063452	RU Mat’-i-machekha	Leaves	Dried	Tea	1			1
**Betulaceae**								
*Alnus* spp. incl. *Alnus glutinosa* (L.) Gaertn. & *Alnus incana* (L.) Moench	RU Ol’kha S Lepp ^A^	Twigs, wood	Fresh	Meat smoking	16	8	5	9
*Betula* spp. incl. *Betula pendula* Roth & *B. pubescens* Ehrh.LE 01063453	RU Bereza S Kask ^A^, kõiv ^B^	Sap	Fresh	Sap	3	10	10	5
			Fermented	Sap	10	11	5	4
			Fermented	*Kvass*	5	1	1	2
			Boiled or pasteurized	Sap			3	
*Corylus avellana* (L.) H.Karst. LE 01063433	S Leshchina, oreshnik (RU)	Wood, twigs	Fresh	Meat smoking			2	
		Nuts	Fresh	Snack	1			
**Brassicaceae**								
*Armoracia rusticana* G.Gaertn., B.Mey. and Scherb. LE 01063403	RU Khrion ^RD^, khren	Leaves, roots	Fresh	Seasoning for fermented cucumbers		1	1	9
		Leaves, roots	Fresh	Seasoning for fermented mushrooms	1		1	4
		Roots	Fresh	Preserve	1	1		
		Root	Fresh	Added to moonshine				1
**Cannabaceae**								
*Humulus lupulus* L. LE 01063406	RU Khmel’	Cones	Fresh	Beer	1	2		
**Caryophyllaceae**								
*Stellaria media* (L.) Vill. LE 01063424	RU Mokritsa	Aerial parts	Fresh	Salad				2
**Cupressaceae**								
*Juniperus communis* L. LE 01063408	RU Veres ^RD^, veresk ^RD^, veresovka ^RD^, mozhzhevel’nik S Kadajas ^B^, kadakas ^A^, katai ^B^	Wood, twigs	Fresh	Meat smoking	11	5	3	3
		Twigs	Fresh	*Kvass* (seasoning, filter)	4	4		
				Seasoning for beer	1			
		Twigs, fruit	Fresh	Seasoning for birch sap		2		
		Fruit	Fresh, dry	Seasoning for bread		1		1
				Seasoning for cabbage			1	
				Seasoning for food (pilaf)			1	
				*Suslo*		1		
			Dried	Tea	2			
**Cyperaceae**								
*Schoenoplectus lacustris* (L.) Pallas	RU Trosta ^RD^, trostnik, kamysh	Bottom of stalks	Fresh	Snack		2		
**Elaeagnaceae**								
*Hippophae rhamnoides* L.	RU Oblepikha	Leaves	Dried	Tea				1
**Equisetaceae**								
*Equisetum arvense* L. LE 01063431	RU Khvoshch; pupyshi ^RD^, papushi ^NF^	Strobilus bearing stems	Fresh	Snack	4	9		
**Ericaceae**								
*Calluna vulgaris* (L.) Hull LE 01063447, dsPCH19-005	S Veres (RU) ^RD^	Twigs with flowers	Dried	Tea	1			
*Empetrum nigrum* L.	RU Voronets ^NF^	Fruit	Fresh	Snack				1
*Vaccinium myrtillus* L. LE 01063440, dsPCH19-025	RU Chernika S Mustik’ ^B^, must’kas ^NF^, mustikas ^A^	Fruit	Fresh	Snack	1		4	
				Dessert (crushed with sugar)				1
				Raw jam		4	1	2
			Boiled	Kompot			1	1
				*Mors*			1	1
				*Kissel*	1	1	1	3
				Jam	5	10	22	9
				Pie filling	3	4	5	15
			Dried	Dried preserve	1			
			Frozen	Snack			12	9
		Aerial parts	Dried	Tea	2	2	1	3
*Vaccinium oxycoccos* L. LE 01063435	RU Kliukva, zhuravina ^RD^ S Jõhvikas ^A^, kurõmari ^NF^, kuremari ^B^	Fruit	Fresh	Snack	1	1	4	4
				Condiment for meat	1			
				Raw jam	1	2	3	4
				Seasoning for fermented cabbage	2	5	5	4
				Pie filling	3	1	2	3
				Added to vodka				1
			Soaked in water	Preserve	1	2	5	4
			Fermented	*Suslo*		1		
			Boiled	*Mors*		4	4	6
				*Kissel*	3	3	2	2
				*Mousse*	3	3	3	2
				Jam	5	2	11	7
			Frozen	Preserve			9	2
*Vaccinium ulginosum* L. LE 01063439	RU Golubika, gonobol’ S Sinikas ^A^, joovikas ^B^, johvik ^NF^	Fruit	Fresh	Snack		2	2	1
				Pie filling	1			
			Boiled	Mors			1	
				Jam		1	5	1
			Fermented	Wine		1		
			Frozen	Frozen preserve				1
*Vaccinium vitis-idaea* L. LE 01063412	RU Brusnika S Palohka ^B^, palohkas ^B^, pohlakas ^A^	Fruit	Fresh	Snack	1	2	3	1
				Soaked berries	3	4	6	8
				Raw jam		1		2
				Liquor	1			1
			Soaked	Seasoning for meat or potatoes	1	1		1
			Boiled	Kompot				1
				*Mors*		3	3	3
				*Kissel*				2
				Jam (often mixed with apples)	6	7	17	10
				Pie filling (often mixed with apples)	2	1	8	12
			Frozen	Frozen preserve			8	4
		Leaves	Dried	Tea	3	3		
**Leguminosae**								
*Caragana arborescens* Lam. LE 01063449	RU Akatsiia	Pods	Fresh	Snack		3		
*Trifolium montanum* L. LE 01063393, dsPCH19-009, dsPCH19-021	RU Gornyi klever ^RS^, belyi klever, romashka-kashka ^NF^, gornaia romashka ^NF^, medunitsa ^NF^ S Tsäihain ^NF^	Inflorescences	Dried	Tea	2	6	2	1
*Trifolium* spp., incl. *Trifolium pratense* L. LE 01063455, LE 01063456, dsPCH19-024	RU Klever, krasnyi klever	Inflorescences	Dried	Tea			1	2
*Trifolium repens* L. LE 01063437, LE 01063415	RU Kashka	Inflorescences, nectar	Fresh	Snack	5			
**Fagaceae**								
*Quercus robur* L. LE 01063451	RU Dub S Tamm ^A^	Leaves	Fresh	Seasoning for fermented cucumbers		1	3	5
				Seasoning for fermented mushrooms				1
		Acorns	Fresh or baked	Snack		2		
		Bark	Fresh	Added to moonshine	2			
		Wood, twigs	Fresh	Meat smoking				1
**Grossulariaceae**								
*Ribes nigrum* L.	RU Chiornaia smorodina	Buds	Fresh	Snack		1		
		Leaves	Fresh	Seasoning for fermented cucumbers	2	1	9	13
			Fresh	Seasoning for fermented mushrooms	2		5	9
			Fresh	Seasoning for birch sap			3	
			Fresh	Seasoning for *kvass*				1
			Dried	Tea	3		6	13
**Hypericaceae**								
*Hypericum perforatum* L. LE 01063443, LE 01063428, dsPCH19-007, dsPCH19-018	RU Zveroboi S Naistepuna ^A^	Aerial parts	Dried	Tea	3	5	13	10
**Lamiaceae**								
*Lamium album* L.	RU Belaia krapiva, medunitsa ^NF^	Flowers	Fresh	Snack		1		
*Mentha* spp. dsPCH19-001	RU Miata, perechnaia m. ^RS^, m. polevaia ^RS^, m. lesnaia ^NF^, m. beregovaia ^NF^, m. dikaia ^RD^ S Münt ^A^, piparmünt ^A^, mjatad ^ER^	Aerial parts	Dried	Tea	5	7	14	12
*Mentha arvensis* L. LE 01063473								
			Fresh	Seasoning for drinks: birch sap or Kompot			1	
			Fresh or dried	Seasoning for meat			1	
*Origanum vulgare* L.dsPCH19-008, dsPCH19-003, dsPCH19-017	RU Dushitsa	Aerial parts	Dried	Tea	1	1	4	7
			Tincture	Added to moonshine				1
*Prunella vulgaris* L.	RU Gorlianka ^RD^	Aerial parts	Dried	Tea				1
*Thymus serpyllum* L.	RU Chabrets, bogorodichnaia trava ^NF^, bogoroditskaia trava ^RD^, ivanova trava ^NF^ S Jaanihaina ^NF^	Aerial parts	Dried	Tea	2	1	1	2
			Fresh	Seasoning for mushrooms				1
**Malvaceae**								
*Tilia cordata* Mill. LE 01063409, dsPCH19-022, dsPCH19-032	RU LipaS Pähn ^B^, pähnapuu ^B^	Flowers	Dried	Tea	9	4	4	3
		Leaf buds	Fresh	Snack	2	4		
		Sap	Fermented	Fermented sap	1			
**Onagraceae**								
*Epilobium angustifolium* L. dsPCH19-015	RU Ivan-chai, kiprei, koporskii chai, konevnik ^RDX^	Leaves, flowers	Dried, fermented	Tea	2	1	11	8
**Oxalidaceae**								
*Oxalis acetosella* L. LE 01063434	RU Zaiach’ia kislitsa ^RD^, zaiach’ia kapusta ^RD^, zaiachii shchavel’ ^NF^, kislitsa, kukushkin glaz ^NF^ S Jänesekapsas ^A^, käosilm ^NF^	Leaves	Fresh	Snack	10	6		
			Boiled	Soup			2	
**Pinaceae**								
*Pinus sylvestris* L.	RU Sosna S Petäi ^B^, mänd ^A^	Spring shoots, needles	Fresh	Snack	2	1		1
		Cones	Boiled	Jam			1	
		Cambium	Fresh	Snack	1			
**Plantaginaceae**								
*Plantago major* L. LE 01063457	RU Podorozhnik	Leaves	Fresh	Salad				1
**Poaceae**								
*Elymus repens* (L.) Gould	RU Pyrei	Stalks, juice	Fresh	Snack		1		
Poaceae	RU Trava (lit. ‘grass’)	Stalks, juice	Fresh	Snack	2	1		
**Polygonaceae**								
*Polygonum aviculare* L. LE 01063454, LE 01063423	RU Gorets ptichii	Aerial parts	Fresh	Seasoning for fermented cucumbers				1
*Rumex acetosa* L. LE 01063414	RU Shchavel’. kiselka ^RD^, kislitsa ^RD^ S Hapuhain ^B^, hublikas ^NF^ (EE)	Leaves	Fresh	Snack	6	8	1	
				Salad				1
			Boiled	Soup	8	10	13	12
			Cooked	Pie filling		1		1
			Salted	Preserve	1	1		1
			Frozen	Preserve			1	2
**Primulaceae**								
*Primula veris* L. dsPCH19-027	RU Petushki ^RD^, pervotsvet, primula	Leaves, flowers	Fresh	Salad				1
		Leaves, flowers	Fresh	Snack		1		
**Rosaceae**								
*Amelanchier* spp.	S Irga (RU)	Fruit	Fresh	Snack	1			
*Crataegus* spp. (incl. *Crataegus submollis* Sarg. LE 01063511)	RU Boiaryshnik	Fruit	Dried	Tea				2
*Filipendula ulmaria* (L.) Maxim.	RU Tavolga	Flowers	Dried	Tea				1
*Fragaria vesca* L. LE 01063496	RU Zemlianika S Metsmaasikad ^A^, metsmaasikas ^A^, mõtsmaasik ^B^, metsmaasik ^A^, maasikas ^A^, maask ^B^	Fruit	Fresh	Snack	1	2	2	3
				Raw jam			3	1
			Boiled	Kompot				1
				Jam	1	1	7	6
				Pie filling	2		1	2
				*Mousse*				1
			Frozen	Preserve			5	3
		Leaves	Dried	Tea	1	1	1	3
*Prunus padus* L.	RU Cheremukha S Toomingas ^A^ (EE)	Fruit	Fresh	Snack	5	5	1	
			Fresh	Pie filling		1		
			Tincture	Added to moonshine	1			
*Rosa* spp. dsPCH19-030, dsPCH19-014	RU Shipovnik S Kibuvits	Fruit	Dried	Tea	1	1		4
*Rubus caesius* L., *Rubus nessensis* Hall	RU Ezhevika, kumanika ^RD^	Fruit	Fresh	Snack	2	1	3	4
				Raw jam				1
				Pie filling				1
			Boiled	Kompot				
				Jam		1		
			Frozen	Frozen preserve				2
		Twigs	Dried	Dried (especially in winter)	2			
*Rubus chamaemorus* L.	RU Moroshka S Murakas ^A^, murahka ^B^, murahk ^B^	Fruit	Fresh	Snack	3	3	2	2
				Soaked berries				1
				Raw jam	1		1	1
				Pie filling	1		1	2
			Boiled	Kompot			1	1
				Jam, jelly	5	2	11	2
				*Mousse*			1	
			Frozen	Frozen preserve			5	2
		Sepals	Dried	Tea			1	
*Rubus idaeus* L.	RU Malina S Vabarna ^A^, vabarnas ^A^, vabarn ^A^ EE Vaarikas ^A^	Fruit	Fresh	Snack	1		1	2
				Raw jam			1	2
				Pie filling	3		2	5
			Boiled	Jam	2	1	14	11
				Kompot				3
				*Kissel*	1			1
			Fermented	Wine				2
				Christmas drink			1	
			Fresh	Added to moonshine		1		
			Frozen	Preserve			8	4
		Twigs, sometimes twigs with berries	Dried	Tea (especially in winter)	4	1	5	5
*Rubus saxatilis* L.	RU Kostianika	Fruit	Fresh	Snack				2
			Frozen	Frozen preserve				1
*Sorbus aucuparia* L. LE 01063446	RU Riabina S Pihlapuu ^A^	Fruit	Fresh (frozen)	Snack	3	1	3	
				Candied fruit		1		
				Added to moonshine				1
			Boiled	Jam				1
				Kompot			1	
				*Kissel*		1		
				Pie filling				1
				Condiment for meat			1	
			Fermented	Wine	2			1
				Christmas drink			1	
			Dried	Tea	1			
**Salicaceae**								
*Populus alba* L.	RU Topol’	Leaves	Fresh	Snack		1		
*Populus tremula* L. LE 01063429	S Osina (RU)	Wood, twigs	Incense	Meat smoking	1			
**Sapindaceae**								
*Acer platanoides* L. LE 01063411	RU Klion S Vaher ^A^	Sap	Fresh	Fresh sap	3	6	5	5
				Added to birch sap		1		
			Fermented	Fermented sap	4	2		
				*Kvass*	1	1	1	
			Boiled	Sterilized sap				1
		Seeds	Fresh	Snack		1		
**Urticaceae**								
*Urtica dioica* L. LE 01063436	RU Krapiva S Nõgõss ^B^	Aerial parts (young plant)	Fresh	Snack	1	1		
				Salad		1		2
			Boiled	Soup	7	11	2	3
			Baked	Bread additive		1		
			Dried	Tea			1	
			Fresh	Meat smoking	1			1
Unidentified taxon	RU Barkannik ^NF^, morkovnik	Aerial parts	Fresh	Snack		1		
			Cooked	Seasoning for meat		1		

(RU) indicates that all Seto participants only provided a Russian name. (EE) indicates that the participants clearly indicated that the name is Estonian and not Seto. Plant name analysis based on [52,53,54,55]: ^A^ = plant name spread all across Estonia, ^B^ = dialect plant name (Seto and Võro), ^NF^ = new plant name from field work, ^ER^ = Seto name borrowed from Russian, ^RD^ = Russian dialect name, ^RDX^ = Russian dialect name outside NW region. Common Russian names are not indicated with superscript. *Kissel*—a viscous drink made from berries with the addition of starch, the more ancient form was made with oats; *kompot*—a sugary drink made from fruits or berries and sometimes stored for winter; *kvass*—a fermented drink made from water or birch sap with the addition of barley malt breads or, more recently, rye bread or industrial concentrate; *mors*—a drink made from fresh berries that is consumed immediately after mixing or is brought to a boil; mousse—a dessert made from whipped boiled semolina with the addition of red berries, which was popular in the 1960s–1980s (some participants claimed to have learned to make it in Estonian culinary schools); *suslo*—a sweet soft drink made from barley malt or flour.

**Table 5:** Jaccard Similarity Index (JI) calculated for taxa used by Estonians (EE), Estonian Setos (EE Setos), Russian Setos (RU Setos) and Russians (RU)–Current uses–Past uses–All uses. Right top of each table: all taxa. Left bottom of each table: taxa recorded three or more times.

**Current, ≥3 taxa**	**Current, All Taxa**				
	**JI**	**EE**	**EE Setos**	**RU Setos**	**RU**
	**EE**	**X**	**68.29**	**54.17**	**49.12**
	**EE Setos**	**73.68**	**X**	**65.12**	**46.43**
	**RU Setos**	**54.55**	**65.85**	**X**	**61.11**
	**RU**	**52.17**	**55.56**	**80.00**	**X**
**Past, ≥3 taxa**	**Past, All Taxa**				
	**JI**	**EE**	**EE Setos**	**RU Setos**	**RU**
	**EE**	X	85.71	57.41	60.00
	**EE Setos**	83.72	X	**57.41**	57.14
	**RU Setos**	64.58	**64.58**	X	69.64
	**RU**	61.22	61.22	90.48	X
**All time, ≥3 taxa**	**All Time, All Taxa**				
	**JI**	**EE**	**EE Setos**	**RU Setos**	**RU**
	**EE**	X	69.49153	58.46154	53.33333
	**EE Setos**	88.88889	X	**59.01639**	49.35065
	**RU Setos**	37.93103	**61.53846**	X	64.70588
	**RU**	58.18182	58.49057	91.11111	X

**Table 6:** Wild food plant names recorded from Setos in Russia and Estonia.

	Russia			Estonia		
	Number of Plant Names	Number of Informants Who Provided Names in Corresponding Language	Number of Taxa	Number of Plant Names	Number of Informants Who Provided Names in Corresponding Language	Number of Taxa
Dialectal (Seto/Võro)	25	18	16	73	37	37
Russian	72	24	66	1	2	1
Spread all over Estonia	27	18	19	73	37	51
Total	-	25	66	-	37	62

## Data Availability

The datasets generated for this study are available from the authors upon reasonable request. Data from ERC project DiGe will be fully available after the project ends.
